# From reflexivity to collectivity: Challenging the benevolence narrative in global health

**Published:** 2017-04-20

**Authors:** Lori Hanson

**Affiliations:** 1 Department of Community Health and Epidemiology, University of Saskatchewan, Saskatchewan, Canada

*If you have any sense of responsibility, at all, stay with your riots here at home… you will know what you are doing, why you are doing it, and how to communicate with those to whom you speak. And you will know when you fail. If you insist on working with the poor, if this is your vocation, then at least work among the poor who can tell you to go to hell… I am here to challenge you to recognize your inability, your powerlessness and your incapacity to do the “good” which you intended to do.*^1^

In his now classic 1968 speech to a group of students embarking on a volunteer summer experience in Mexico, critical educator and priest Ivan Illich suggests that students do away with their pretense. “To hell with good intentions” he proclaims. It was a scathing indictment of the first modern era of international volunteer do-gooders, and arguably as relevant today as then. Like volunteerism, the premise that underlies global health practice and its accompanying curricula in many faculties of medicine is frequently that of good intention, of benevolent service. Yet, as did Illich, an increasing number of medical educators and global health scholars are interrogating those practices, and their underlying assumptions, accompanying curricula and nature of engagements; they are troubling the benevolence narrative. Not all, but a number of the articles and essays in this CMEJ supplement and the earlier special edition on globalization and health suggest a certain uneasiness. Such discomfort with the field and open discussion of its contradictions is, in my view, healthy, timely, and apt. Good scholarship takes itself as an object of scrutiny, and so, let us take a moment to question our premise and to ask -- what is missing?

The thoughtful writing in these two issues is fairly representative of global health education, research, and practice as a field largely located in bio-medical and behavioural paradigms. As with the field more broadly, multi-disciplinary social determinants of health approaches or framings are largely absent from this set of articles. Perhaps that is partly why, when it exists, the articles’ critiques of globalization are vague. Seldom is the term defined or analyzed.

The intellectual task of relating how it acts as a “structural driver”^2^ of health inequity is largely skirted. Instead, globalization frequently sits as a background or a shadow, an inevitability, a constantly evolving, but rather invisible force. Practices and policy conundrums are described as if sitting atop globalization. Yet something is wrong in this highly unequal, globalized world. And that too is evident. Many articles thus end on a call for social justice. But again, the notion is not explored, at least not with the verve that reflective practice is -- or the two get conflated. It seems that personal reflection will somehow contribute to social change or work to resolve power inequities, but those connections are not explicitly made. In these articles there appears to be little on the relevance, or perhaps capacity to comprehend, complex global health problems as embedded in structural injustices. The notion that one might approach the question of ‘what is to be done’ in global health as an outgrowth of a big picture analysis seems largely unexplored.

Reflexivity seems a predominant resolution to the paradoxes of global health. A few articles suggest the importance of reflection on white privilege; one describes a classroom exercise to openly discuss personal experiences of racism. One does step back and proposes the concept of a global health “voluntariat,” embedding the contradictory positioning of the modern student of global health in a neoliberal world. These are all important matters.

Yet I would submit that we might do well to bookend the personal reflexivity or reflective practice so eloquently argued for in various articles herein, with the collective responsibility to study, comprehend and enact the principles of social justice, health equity and solidarity by understanding, first, not only ourselves, but the systems in which we are all embedded, and the politics that allows those systems to function. And that we could then start to move self-critical analyses beyond an individual response to a collective acknowledgement or “collectivist account”^3^ of global systems of power and injustice. A collective account would entail recognition - that high income countries (HICs) wouldn’t exist without low and middle income countries (LMICs), that the privilege of some depends on other’s lack thereof, that “we” and “they” are contiguous and interconnected, that our economic and political systems intertwine and interdepend in highly structured and very unequal ways; that these systems have names and histories and that it matters, too, that we discuss them. Such an account may also require holding up a different kind of mirror on our academic work. Our lack of recognition of universities’ more or less assimilationist roles in the capitalist economic system and in ongoing historic processes of colonialism into which international global health educational interventions insert themselves - and in which we all participate unintentionally or not - is part of the problem. Perhaps it is time we acknowledge that there is no innocence, that benevolent intention is blinding, and that we might do well to abide by Illich, and stop wasting precious time and energy searching for it.

Given that we are discussing this in the CMEJ, we might, alternatively - or additionally - contemplate how our own government and elites engage in the very same neoliberal globalization in ways that both foment and exacerbate health inequities. For example, what does it mean that Canada is an extractivist state - home to headquarters of two-thirds of the world’s mining companies - in terms of the kinds of environmental damages, labour law repeals, and community conflicts that have erupted in Latin America over the past 15 or so years?^4^ How might that reality affect the health of hundreds of thousands of people - particularly indigenous peoples - in Latin America? How might the Canadian government’s sale of tanks to the brutal Saudi Arabian regime affect health there?^5^ How did Canada’s long history of refusal to ban chrysotile asbestos through the Rotterdam Convention affect the health of millions of workers worldwide?^6^,^7^ And what of the effects of Harper era policies such as the repeal of the legislative restraints on the selling of uranium to a country (India) that has not signed the nuclear non-proliferation treaty?^8^ And how ought this Canadian pattern - prioritizing the sale of commodities for profit over concerns with international treaties and obligations; over health and human rights; over sovereignty in indigenous communities - affect our scholarship in the realm of globalization and health? Perhaps it is time to turf the “national mythology”^9^ of innocence, that we are nice Canadians, that we are benevolent travellers. Perhaps we could seek to write articles that reflect a framing of global health concerns and educational interventions in terms that address not merely individual roles and technical capacities, but the contradictions and hypocrisies of the Canadian “white burden” on the health of marginalized communities – including indigenous communities at home?

It is ever so much easier to ignore history, to reflect on personal practices, or to look afield for deficiencies than it is to face the great collective mirror where we would see our own government, institutions, elites and industries propagating the conditions that lead to health inequities. We live in dangerous times and the global health community cannot afford to ignore the politics enveloping international practices in which we individually and collectively engage.

More than ever, the West’s unresolvable moral “quest for innocence in a post-colonial world”^10^ needs to be put to rest. I think we can do more, guided not only by introspection, humility, and reflexivity, but also by a practice of solidarity and social justice and a rigorous critique of the systemic issues into which our work is inserted. There are useful educational interventions and technical practices in the field of global health, but it is time to let the underlying good intentions go, replacing them with a collective account from which other kinds of collective actions - on health and justice - rise up.
